# Visual Estimation of Bacterial Growth Level in Microfluidic Culture Systems

**DOI:** 10.3390/s18020447

**Published:** 2018-02-03

**Authors:** Kyukwang Kim, Seunggyu Kim, Jessie S. Jeon

**Affiliations:** 1 Robotics Program, Korea Advanced Institute of Science and Technology, 291 Daehak-ro, Daejeon 34141, Korea; kkim0214@kaist.ac.kr; 2 Department of Mechanical Engineering, Korea Advanced Institute of Science and Technology, 291 Daehak-ro, Daejeon 34141, Korea; ksg5825@kaist.ac.kr; 3 KAIST Institute for Health Science and Technology, 291 Daehak-ro, Daejeon 34141, Korea

**Keywords:** Lab-on-a-chip, 3D Printing, microfluidics, solid modeling

## Abstract

Microfluidic devices are an emerging platform for a variety of experiments involving bacterial cell culture, and has advantages including cost and convenience. One inevitable step during bacterial cell culture is the measurement of cell concentration in the channel. The optical density measurement technique is generally used for bacterial growth estimation, but it is not applicable to microfluidic devices due to the small sample volumes in microfluidics. Alternately, cell counting or colony-forming unit methods may be applied, but these do not work in situ; nor do these methods show measurement results immediately. To this end, we present a new vision-based method to estimate the growth level of the bacteria in microfluidic channels. We use Fast Fourier transform (FFT) to detect the frequency level change of the microscopic image, focusing on the fact that the microscopic image becomes rough as the number of cells in the field of view increases, adding high frequencies to the spectrum of the image. Two types of microfluidic devices are used to culture bacteria in liquid and agar gel medium, and time-lapsed images are captured. The images obtained are analyzed using FFT, resulting in an increase in high-frequency noise proportional to the time passed. Furthermore, we apply the developed method in the microfluidic antibiotics susceptibility test by recognizing the regional concentration change of the bacteria that are cultured in the antibiotics gradient. Finally, a deep learning-based data regression is performed on the data obtained by the proposed vision-based method for robust reporting of data.

## 1. Introduction

Measurement of bacterial cell number is a critical process during bacterial cell culture for clinical diagnosis as well as the antibiotic susceptibility test (AST) [1,2,3]. While a number of methods for cell density measurement, including visible spectrometry (VIS), cell counter (CC), and fluorescence activated cell sorting (FACS), have been developed, the optical density (OD) method, which measures the absorbance/scattering of the media containing bacterial cell particle, is widely used among the different methods due to its advantages, which include its fast measurement time and simple procedure [4]. However, the OD method is limited in usage with microfluidic platforms, as most commercial OD measurement devices are not designed to detect the absorbance level of small volumes of culture broth (<1 mL) [4].

The size and capacity of the culture vessel varies from 1 L-sized batch culture reactors to 1 mL-volume 48-well flower plates, depending on the purpose and scale of the culture experiment [5]. However, recent advances in bacterial culture techniques have shown that robust performance of bacterial growth can also be monitored for microfluidic culture devices at a micro/nanoliter-scale volume [6]. Furthermore, the recent development and expansion of various Lab-on-a-Chip (LoC) devices for bacterial culture platforms increase the need for growth measurement methods on LoC devices at small volume culture scales [7]. The colony-forming unit (CFU) counting [8] or microcolony area measurement may be used, but these methods are limited, as they cannot generate immediate measurement outputs. On-chip microfluidic droplets allow high-throughput single-cell level counting [9]; however, this requires microfluidic setups and additional devices that are usually not compatible with the well-known OD devices.

Recently, bacterial growth estimation has been performed for a macro/millifluidic scale culture using a fast Fourier transform (FFT)-based imaging method, as previously reported by our group [10,11]. The FFT method was used to measure the magnitude of the marker concealment, and the blurring of the broth due to cell growth was easily detectable [12]. Similarly, based on the observation that the microscopic image was planar and clear at the early stage of the culture, and became speckled as the number of bacterial cell increased during the culture, we here present a new technique for estimating the growth level of the bacteria in the microfluidic scale by applying the FFT principle. The following sections describe the experimental setup of the bacterial culture in the proposed microfluidic device, procedures for acquiring FFT values from the microscopic image, and checking the relations of the FFT-measured value under various conditions. Then, the measurement values obtained using the conventional and the proposed methods are compared. Furthermore, an application of the proposed FFT estimation method is shown in monitoring AST in microfluidic devices. Finally, we present the deep neural network (DNN)-assisted preliminary results of growth measurement estimation, which were obtained by using DNN as a regression method.

## 2. Materials and Methods

### 2.1. Microfluidic Device Preparation

**Figure 1:**
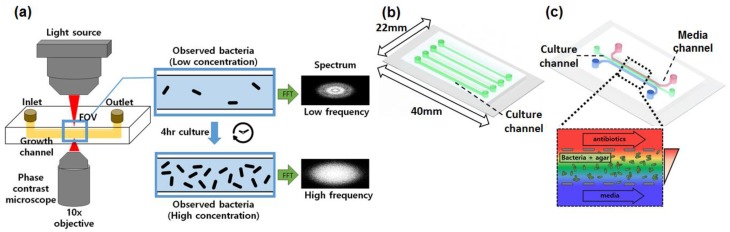
Overall setup of the proposed method and types of microfluidic devices used for bacterial culture. (**a**) Proposed setup and data-flow diagram of the marker-free growth monitoring system; (**b**) Schematic of the liquid broth culture PDMS device. (**c**) Schematic of the agar culture PDMS device. Posts at the boundary of the middle channel separates agar from the lateral channels. Two lateral channels are used for broth supply and chemical gradient formation. The two devices (**b**,**c**) have equal dimensions.

The overall setup of the proposed method and the two types of polydimethylsiloxane (PDMS) microfluidic devices used in this research are shown in Figure 1. The straight channel device was utilized for the liquid suspension culture and the other multi-channel device was used for the bacteria culture on the agar gel with liquid broth on the sides. The liquid culture chip had a straight microchannel with 26.4 mm length and 1.2 mm width (Figure 1b). The design for the agar culture device had been reported previously; a large main channel was placed in the center to hold polymeric materials like agarose or collagen with the cells. The devices were fabricated by following the protocol provided by Shin et al. [13]. Two channels holding the liquid broth were placed on the side of the main channel for a continuous supply of the nutrients and moisture to the main culture channel. The multiple micro-sized posts separated the main channel from the side channels, aiding circulation of the supplied liquid broth while holding the agarose matrix to the channel (Figure 1c). Comparison of the proposed setup with the previous designs is illustrated in Supplementary Figure S1.

### 2.2. On-Chip Bacterial Culture and Image Acquisition

*Pseudomonas aeruginosa* was used for the experiment, and the bacterial cell line was kindly provided by the Nanobiomedicine Laboratory of KAIST. The frozen glycerol stock was thawed and subcultured by using the Tryptic Soy Broth (TSB, BD Biosciences, Franklin Lakes, NJ, USA) in the 37 °C incubator.

The bacterial cells were suspended in 50 mL TSB broth and cultured overnight with the shaking incubator to reach OD_600_ values of 1.0~1.5 before culturing in the microfluidic devices. The bacteria were inoculated into the clean TSB broth with the platinum loop and 10 μL volume from the inoculated broth was injected to the channel of the liquid suspension culture chip.

In the case of the agar culture device, bactoagar (Lab Pharm Service solution, Daejeon, Korea) was added to the TSB broth with a final concentration of 1%. The agar containing TSB was autoclaved and cooled to about 80 °C. 100 μL of the inoculated TSB and 300 μL of the autoclaved TSB were mixed and 10 μL volume of the mixture was injected into the center culture channel of the agar culture chip before the mixture coagulated due to cooling. After 5 min of cooling, the clean TSB was injected into the side channel. The microfluidic devices containing the inoculate media were placed in the 37 °C incubator and used for the further experiments.

### 2.3. Image Acquisition and Processing for Growth Estimation

The cultured chips were placed under the phase contrast microscope to obtain in-channel images. A Zeiss AXIO Observer Z1 microscope (Carl Zeiss AG, Oberkochen, Germany) was used with settings of 100x total magnification, 1.11 μm resolution, and numerical aperture (NA) 0.55 condenser.

The cell concentration of the microchannel images was estimated by using the FFT. 2D discrete Fourier transformation (DFT) was performed using the fft2 function of the Python Numpy package. Each pixel of the obtained 2D spectrum image indicates the strength of a certain frequency domain. To remove the noise (week-strength signals), the 2D spectrum image was passed through a threshold function, and pixel values lower than 127 (half of the maximum pixel value in the OpenCV library) were removed. The thresholding value can be adjusted, but the value of 127 showed fine results in the various setups. Then, the non-zero pixels in the filtered 2D spectrum image were counted and used as a score for the growth level. The numbers of the nonzero pixels are proportional to the inner pixel divergence, but are also related to the image size, so the pixel count values were normalized with the largest pixel count values among the all acquired data (current count/maximum(all count values)).

### 2.4. CFU Measurement

The 100 mm diameter tryptic soy agar (TSA) plates were prepared for the CFU measurement. The liquid broth in the liquid suspension culture chip was suctioned using a pipette and diluted to clear 100 μL TSB broth. In the case of the agar culture chip, the liquid broth in the side channels was removed, and the agarose gel in the main channel was harvested by flushing it with the 100 μL TSB broth. The agarose was homogenized by vortexing the mixture to extract bacterial cells from the agarose matrix. Prepared samples were diluted 10^4^-fold, 10^5^-fold, and 10^6^-fold, and spread onto the TSA plate with the spreader. The plates were incubated overnight, and the number of visible colonies was counted. Two chips were prepared at the same time with the same protocol for each culture type.

### 2.5. Culture under Antibiotics Gradient

The agar culture chip was prepared and inoculated as described in the previous section. After 1 h of incubation, both side channels were linked to the custom syringe pump [14] to deliver both the clear TSB broth and the TSB broth with 2.5 μg/mL gentamycin (Thermo Fisher Scientific, Waltham, MA, USA). An additional syringe pump was also linked to the sink to generate a continuous flow of both broths with the speed of 4 μL/min. This flow generated the antibiotics gradient in the main culture channel due to diffusion from the high concentration site to the low concentration site. The verification of the gradient generating function of the used agar chip is available in the previous literature [15,16]. Microscopic images of the channel after connection of the gradient generating pump were recorded every 15 min.

### 2.6. Deep Nerual Network Setups

The AlexNet structure was used for the FFT data regression. As this architecture was originally used for image classification, the 1000 output softmax layer with cross-entropy loss [17] was replaced with a Euclidean loss layer with one output for the regression procedure. Other structures, like convolution, pooling, and fully connected layers, were conserved. The caffe framework was used for the implementation [18]. The network specifications are shown in Table 1. Every activation layer was ReLU (rectifier linear unit) and the pooling was max pooling.

## 3. Results and Discussion

### 3.1. Growth Level Estimation with the FFT

**Figure 2:**
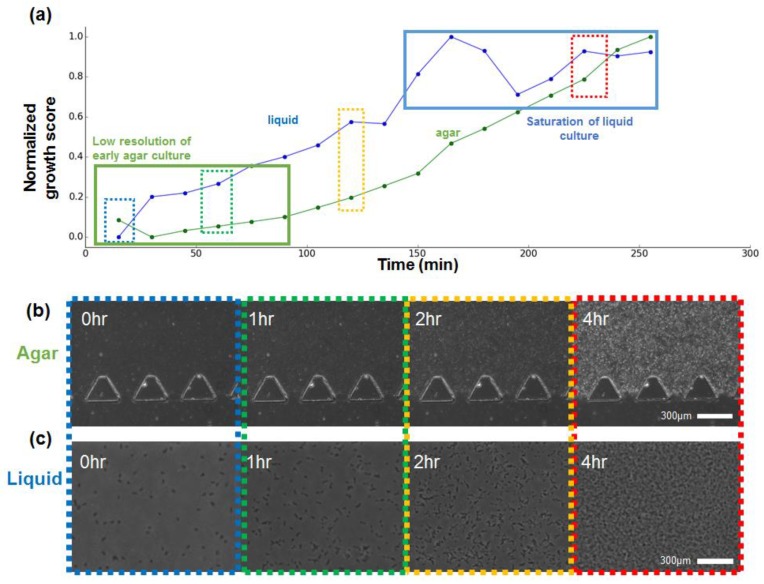
Growth estimation and acquired growth images. (**a**) Growth curve of the liquid culture and agarose culture measured per every 15 min; (**b**) Microscopy image of the agarose culture; (**c**) Magnified microscopy image of the liquid culture.

The FFT-based method was applied to estimate the growth level of the bacterial cells in the microfluidic channel by measuring the increase in pixel difference resulting from an increase of the cell particles visible in the image. The data showing the change in the normalized FFT spectrum pixel count every 15 min from 0 to 4.5 h (total 17 data points from each chip type) are shown in Figure 2a, with the raw microscopic image of the agar culture chip (Figure 2b) and liquid suspension culture chip (Figure 2c). The green line indicates the normalized value of the agar chip image, and the blue line indicates the value of the liquid chip image.

The data shows a constant increase in bacterial growth as time passes, showing that the proposed method can estimate the change of the bacteria in the microfluidic channels regardless of the presence of the agar medium. The liquid culture and the agar culture showed slightly different growth patterns. At the early stage, the liquid culture showed a more rapid increase compared to the agar chip. However, the data from the agar culture chip did show sufficient resolution at the very early stage. The acquired data from 0 to 1.5 h did not show a significant difference (green box in Figure 2a). It is known that bacteria shows faster growth in liquid broth than in a solid agar plate due to nutrient circulation and the easier diffusion of the cell particles. Data patterns similar to the macro environment culture were observed in the microchip culture by using the proposed method. Meanwhile, the growth level of the liquid culture showed faster saturation and low-level resolution at the ending stage of the incubation. The blue box in Figure 2a shows that the growth score of the liquid broth reached the maximum value after about a 3-h period, and that data measured at later time periods did not show a significant difference in value.

Compared to the liquid chip, the agar culture chip showed a steady increase of the score value. The agar chip contains solid media, and a change of the agar gel matrix due to cell growth affects the image. The particles including bacterial cells in the culture channel of the liquid chip float freely, and the particles located out of the focal plane can be observed vaguely during the imaging, resulting in noise during growth estimation by FFT. Overall, the proposed method showed a resolution that was detectable in the range of 15–30 min, regardless of the culture device.

### 3.2. Comparison with Conventional Growth Estimation Methods

In order to confirm the functionality of the proposed method for bacterial growth estimation, the values were compared with one of the conventional methods, the CFU counting method. Previously, we have tried to compare the FFT-based growth estimation to the OD_600_ values, another conventional method for estimating growth level, when the culture vessels were macroscopic and contained sufficient amounts for OD_600_ measurements. Since microfluidic culture chips generally use small sample volumes, which is normally an advantage, the microfluidic platform does not hold enough broth for the OD measurement.

The broth/agar in the culture channel was extracted and spread on the agar plate after dilution. The samples from 0 h and 4 h were used for CFU counting. For the normalized FFT score, a total of 16 microscopic images of the random sights of each condition used in the measurements were obtained from multiple culture devices per condition. Total 12 microscopic images from 2 agar culture devices and 16 images from 6 liquid culture devices. The FFT-based growth score for each image was computed and averaged. The data is shown in Table 2.

An average normalized growth score of 0.115 ± 0.063 was obtained for the 0-h sample of the liquid chip, and a value of 0.875 ± 0.087 was obtained for the 4-h sample of the same chip. The agar culture chip showed 0.079 ± 0.015 at 0 h, and 0.80 ± 0.077 at 4 h. Before the culture, the CFU increased to 70 × 10^6^ CFU/100 μL for the liquid chip and 12 × 10^6^ CFU/100 μL for the agar culture chip after 4 h of incubation. The CFU of the 0-h sample was 2 × 10^5^ CFU/100 μL.

A chi-square test with the null hypothesis that the FFT measurement had no relation with the actual cell concentration (CFU) was performed by setting the CFU as an observation variable and the FFT as an expectation. Both liquid and agar culture showed *p*-values < 0.01, accepting the alternative hypothesis that the FFT measurement correlates with the actual CFU value. Further data related to the CFU calculation are shown in Supplementary Figure S2.

Actual cell culture showed that the proposed method could estimate the growth level. As the number of the live cells in the culture media increases, the growth score measured by the proposed method also increased. The results comparing the tendency of the growth level in the two culture chip types in the previous section showed that the liquid chip had a faster increase rate compared to the agar culture chip. The CFU results showed similar results; although the two chips started at the same inoculation concentration, the colony number obtained from the liquid culture chip showed a much higher number.

### 3.3. Application of the Proposed Technique for the AST Experiment

The antibiotics susceptibility test of the bacterial strain was performed to simulate possible usage of the proposed method in real-world applications. Performing AST on the microchips could increase throughput and efficiency, and the proposed method could gradually increase its performance by accelerating the growth state measurement, which is generally performed by a human operator or CFU measurement.

**Figure 3:**
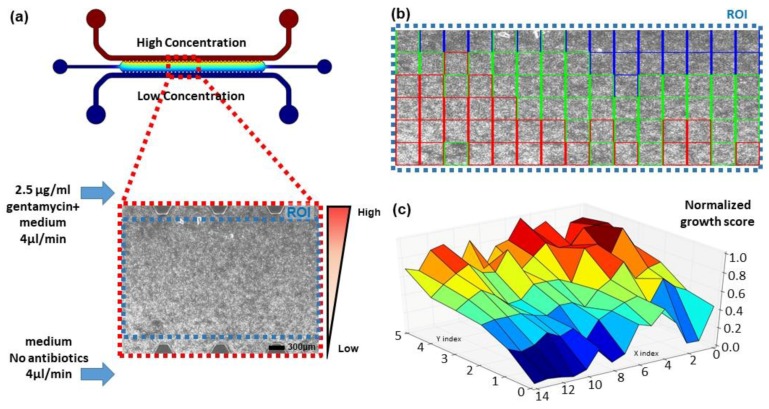
Addition of antibiotics to the system. (**a**) COMSOL CFD simulation image of the used antibiotics gradient generating device and formed biofilm microscopy image; (**b**) FFT regional analysis of the microscopy image. Blue indicates low growth state, green is medium, and red is highly grown biofilm; (**c**) 3D plot of the growth level of the microcopy image. A growth gradient proportional to the antibiotics gradient is formed.

As described previously, the agar culture chip was connected with two syringe pumps to supply two different antibiotic concentration media for generating the antibiotics gradient in the main culture channel of the chip. The channel image was divided into a 6 by 15 equally sized grid, and the growth level of each grid was examined over time (Figure 3). The growth difference of the bacterial cells was observed, as shown by differences in brightness between regions. The regions closer to the high concentration channels showed less brightness compared to the opposite side, where the concentration of the antibiotics was low. The FFT-based normalized growth level estimation was computed, and the grid squares with scores in the top 33% were marked with red, the lowest 33% were marked with blue, and the middle-ranged grids were colored with green in Figure 3b. The color distributions are closely matched to the concentration gradient; blue grid squares are generally located at the top edge, where the antibiotic concentration is high, green squares at the center, and red squares at the opposite edge. A more detailed plot showing the square by square growth level difference is plotted in Figure 3c. The descent from one side to the opposite side proportional to the antibiotics gradient is clearly observable. The effect of the antibiotics on the bacterial cell line was easily detectable with the proposed method, allowing faster automation of the microscale AST process.

**Figure 4:**
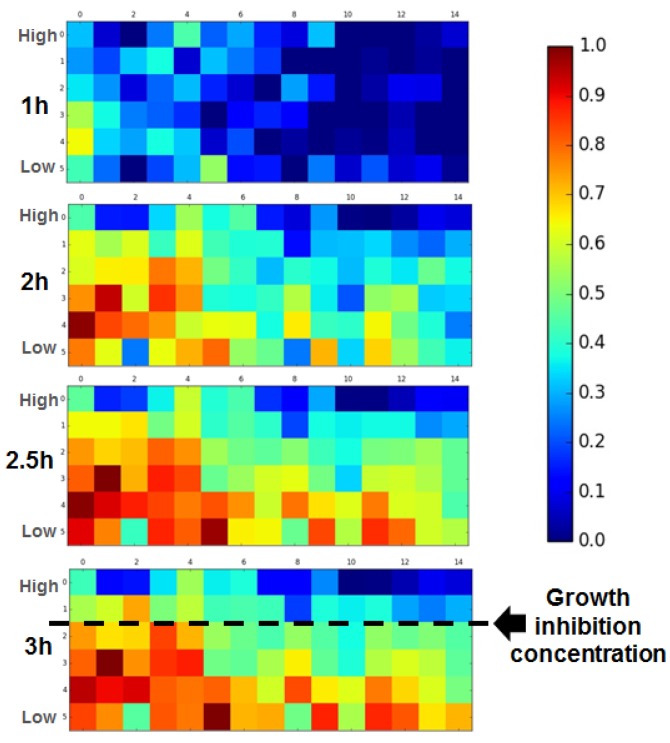
Determination of growth inhibition concentration. Time series FFT regional analysis is performed in the antibiotic gradient generating device microscopy image for MIC determination. Growth is inhibited in the high-concentration region, while growth of bacteria is observable in the low-concentration region.

Knowing whether the antibiotics are susceptible to the bacteria is important, but determining the minimum inhibitory concentration (MIC) [19] is also required for the AST. As the chemical gradient of the antibiotics is linear (the channel image in Figure 3a shows COMSOL computational fluid dynamics simulation results, which indicate the gradient with the color), we can estimate the MIC of the gentamicin is about 1.7 μg/mL, as the bacterial growth is highly suppressed close to the 1/3 mark away from the high-concentration site. Our results are consistent with the range of MIC determined by The Clinical and Laboratory Standard Institute (CLSI). (https://clsi.org/) For more definite data, the growth score of the time-series images were computed and a growth score for each square is shown in Figure 4. Instead of using the values inside one image, the maximum value of the calculation results of all images was selected and used for the normalization. While the overall growth level was low for the 1-h data, the bacteria started to grow as time passed, and the growth level was saturated (no overall change) after 2.5 h of incubation. The color indicating the growth level under the first row changed as time passed, but the grids above the first row remained the same, except for the small differences between the 1-h data and the 2-h data. By examining the time-series image data with the proposed calculation method, more exact MIC values of the antibiotics for a bacterial strain can be quickly examined.

### 3.4. Deep Neural Network Assisted Growth Level Regression

One of the key limitations of the proposed method may be that the measurement relies on direct pixel-wise operation. Anomalies such as image-acquisition noise, bubbles, or foreign materials can affect the frequency level of the image. Sampling algorithms such as sliding window sampling can reduce the effect of these anomalies over the total spectrum, but do not ensure the removal of the noise. Additionally, the maximum and minimum spectrum count values are required for growth score normalization. To remedy these conditions, a deep learning architecture was applied in addition to the image processing system. Recent advances in neural information processing systems have shown great efficiency in image classification and detection [20,21,22,23,24], and data regression is one of the tasks that can be performed using neural networks. In this research, the obtained FFT spectrum image was labelled with the corresponding growth time, and used to train the deep neural network for a better estimation result.

**Figure 5:**
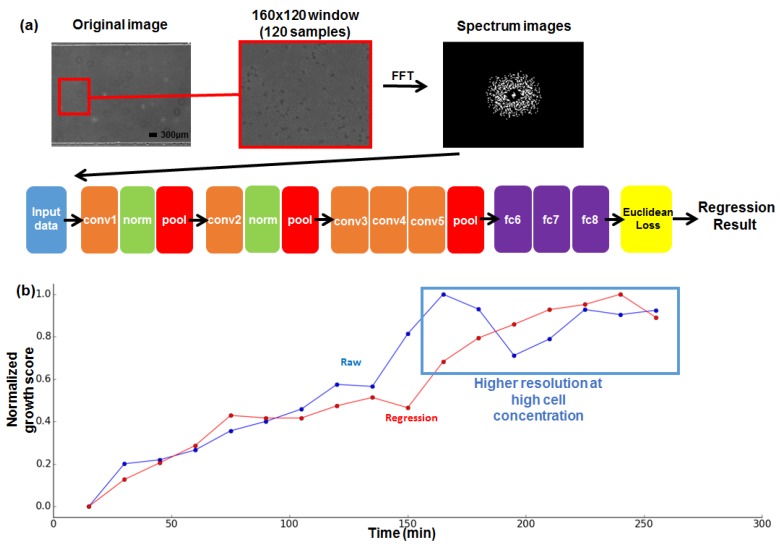
Deep Neural Network (DNN)-based growth estimation. (**a**) Data flow and network structure of the Deep Neural Network-based growth estimation; (**b**) Results of the raw FFT measurement and DNN-based regression result.

A total of 100 images with a size of 160 × 120 pixel were captured from the microscopic image (liquid culture device) at each time step (17 images were used in Figure 3) and converted into spectrum images via FFT. We assumed that the growth level continuously increased during incubation, so 17 labels corresponding to each time stamp were used as a label (output from the DNN). The overall process is shown in Figure 5a.

A randomly selected 20% of the collected images were used as test data. The rest of the images were used as training data. A total of 6000 iterations, with an inverse decaying learning rate of 0.0001, were executed to train the data into the regression network. For better understanding of the neural network, the detailed feed-forward and back-propagation processes are illustrated in Supplementary Figure S3. After the training phase finished, the test data sets were processed by the trained network, and the results with the same labels were collected and averaged to produce a final regression result. For the comparison with the spectrum pixel count score, the regression results were averaged (test results with the same label) and normalized to values between 0 and 1. The regression result is shown in Figure 5b. The network regression output shows a more linear increase compared to the raw FFT pixel count values. In comparison, the FFT result of the liquid culture chip is saturated at the high-cell concentration; the data points after 160 min are indistinguishable due to the saturation value (the blue line in Figure 5b). On the other hand, the regression result (the red line in Figure 5a) shows distinguishable values until almost the last stage (235 min). The significance test for linear regression showed an r2 value of 0. 93 in the case of the raw FFT value, and a value of 0.97 for the deep neural network output, both with *p*-values < 0.01. While both raw pixel counts of the FFT spectrum and the deep neural network regression showed favorable results, deep learning-based regression showed more robust results, closer to its quantized ground truth growth level (original time stamp labelled 1 to 17). The DNN-based regression was tested preliminarily with the liquid culture chip due to its more convenient reproducibility, but we expect the DNN-based regression will work well with the agar chip, as the tendency of the generated FFT spectrum pattern difference is similar, regardless of the culture chip type.

## 4. Conclusions and Future Work

This paper proposed and demonstrated the vision-based method for estimating the bacterial growth level in microfluidic channels, where measurements using the conventional methods are difficult to obtain due to the small volume of the samples used in microfluidic systems. An FFT-based spectrum image was used to detect the increase of the pixel difference in the microscopic image as the cell concentration increased with minute-scale resolution. Furthermore, an AST experiment determining the MIC of the antibiotics was performed with the proposed method to show the possible application of the proposed method. Finally, a deep learning-based data regression for robust data reporting was performed on the data obtained with the vision-based method. Two types of microchip (liquid and agarose) were tested, so appropriate chip types can be used for different target bacteria (e.g., agarose chip for mobile cells like *Escherichia coli*.)

For our future work, we are considering the further application of deep learning to detect and disregard the FFT noise caused by anomalies such as bubbles or foreign materials that often taint microfluidic experiments. Furthermore, we hope that applying advanced deep learning architectures such as one-shot learning [25] may enhance the handling of the irregular shapes of foreign materials, and that upgrading the gradient chips to parallel or 3D structures [26,27] would enable higher throughput of bacterial culture monitoring. Additionally, reducing normalization of the spectrum count step and fully automating the pipeline is under consideration.

## Figures and Tables

**Table 1:** Specifications of the main layers of the deep neural networks used. The number next to the conv layers refers to convolution filter and kernel size. The number next to pool layers refers to kernel and stride size. The number next to the fc layers refers to innerproduct size.

Layer Name	Size	Specification
conv1	96, 11	Filter, kernel
pool1	3, 2	Kernel, stride
conv2	256, 5	
pool2	3, 2	
conv3	384, 3	
conv4	384, 3	
conv5	256, 3	
pool5	3, 2	
fc6	4096	Innerproduct
fc7	4096	
fc8	1000	

**Table 2:** Comparison between cell concentrations estimated by the number of colonies formed during the CFU measurement and calculated FFT score of different samples sources.

Source	CFU (Unit/100 μL)	Normalized FFT Score
Liquid 0 h	2 × 10^5^	0.115 ± 0.063
Liquid 4 h	70 × 10^6^	0.875 ± 0.087
Agar 0 h	2 × 10^5^	0.079 ± 0.015
Agar 4 h	12 × 10^6^	0.80 ± 0.077

## Supplementary Materials

The following are available online at http://www.mdpi.com/1424-8220/18/2/447/s1, Figure S1: (**a**) Structure of marker-based growth monitoring of millifluidic devices proposed by Kim et al. [1]. (**b**) Conventional setups for microfluidic bacteria culture and microscopy. No space for the markers are available due to phase-contrast microscope setups. Figure S2: Fluorescence image of fluorescein (Green, MW = 332.31 g/mol) diffusing into the agar gel from source channel. The flow rate of both media channel was 4 µL/min, and the image was taken 60 min after introducing the dye. The concentration of Fluorescein was 10 µg/mL and the exposure time was 65 ms. Figure S3: (**a**) Barplot showing median and standard error values of FFT count results of 0 h and 4 h (12, 16 samples). Red bars indicate results of the liquid culture and the yellow bars indicate the agar culture. (**b**) CFU counting results for liquid and agar culture at 0 h and 4 h. Figure S4: (**a**) Explanatory image showing feed-forward inference of the Deep Convolutional Neural Network. (**b**) Explanatory image showing the weight update of the neural network for decreasing error between the real value and inferenced output. Important terms used in the main article are marked in red.
