# Supplementary material for: Visual Estimation of Bacterial Growth Level in Microfluidic Culture Systems
Source: Sensors (Basel). 2018 Feb 3;18(2):447. doi: 10.3390/s18020447 (PMC5855051; doi:10.3390/s18020447)
Supplement: Supplementary file 1 [file sensors-18-00447-s001.pdf]

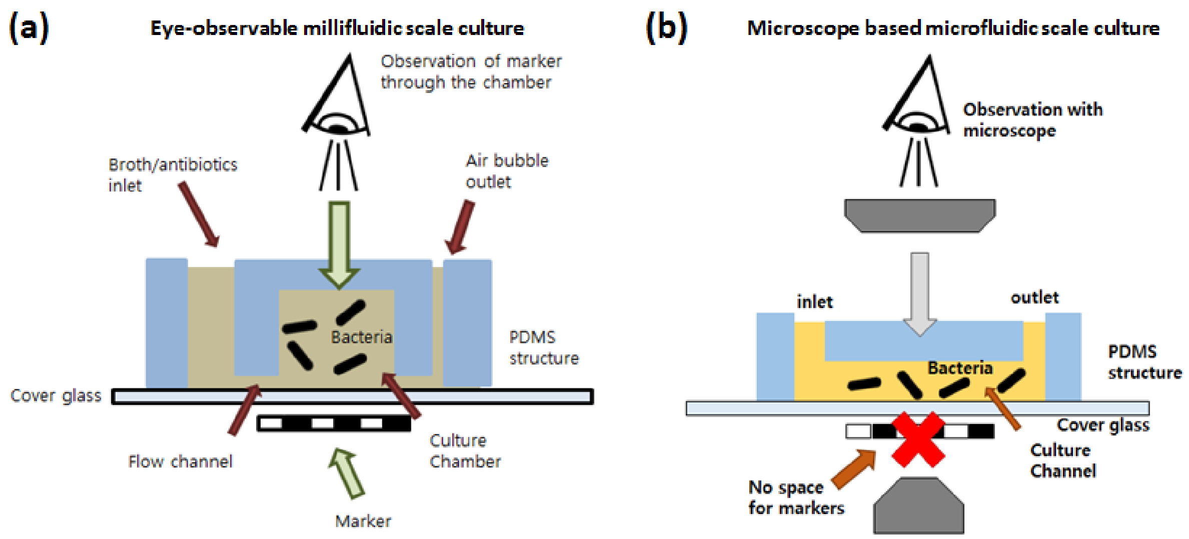

Supplementary Figure 1. (**a**) Structure of marker-based growth monitoring of millifluidic device proposed by Kim et al. [1] (**b**) Conventional setups for microfluidic bacteria culture and microscopy. No space for the markers are available due to phase-contrast microscope setups.

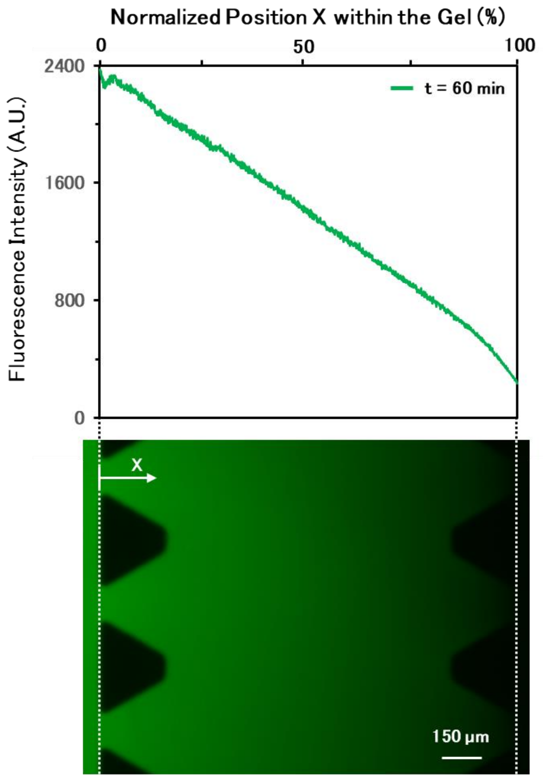

Supplementary Figure 2. Fluorescence image of fluorescein (Green, MW=332.31g/mol) diffusing into the agar gel from source channel. The flow rate of both media channel was 4μL/min, and the image was taken at time 60 minutes after introducing the dye. The concentration of Fluorescein was 10μg/mL and the exposure time was 65ms.

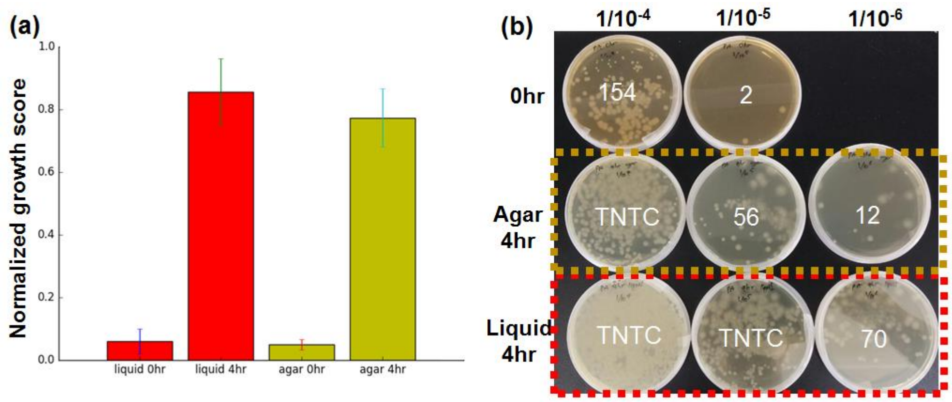

Supplementary Figure 3. (**a**) Barplot showing median and standard error values of FFT count results of 0 h and 4 h (12, 16 samples). Red bars indicate results of the liquid culture and the yellow bars indicate the agar culture. (**b**) CFU counting results for liquid and agar culture at 0 h and 4 h.

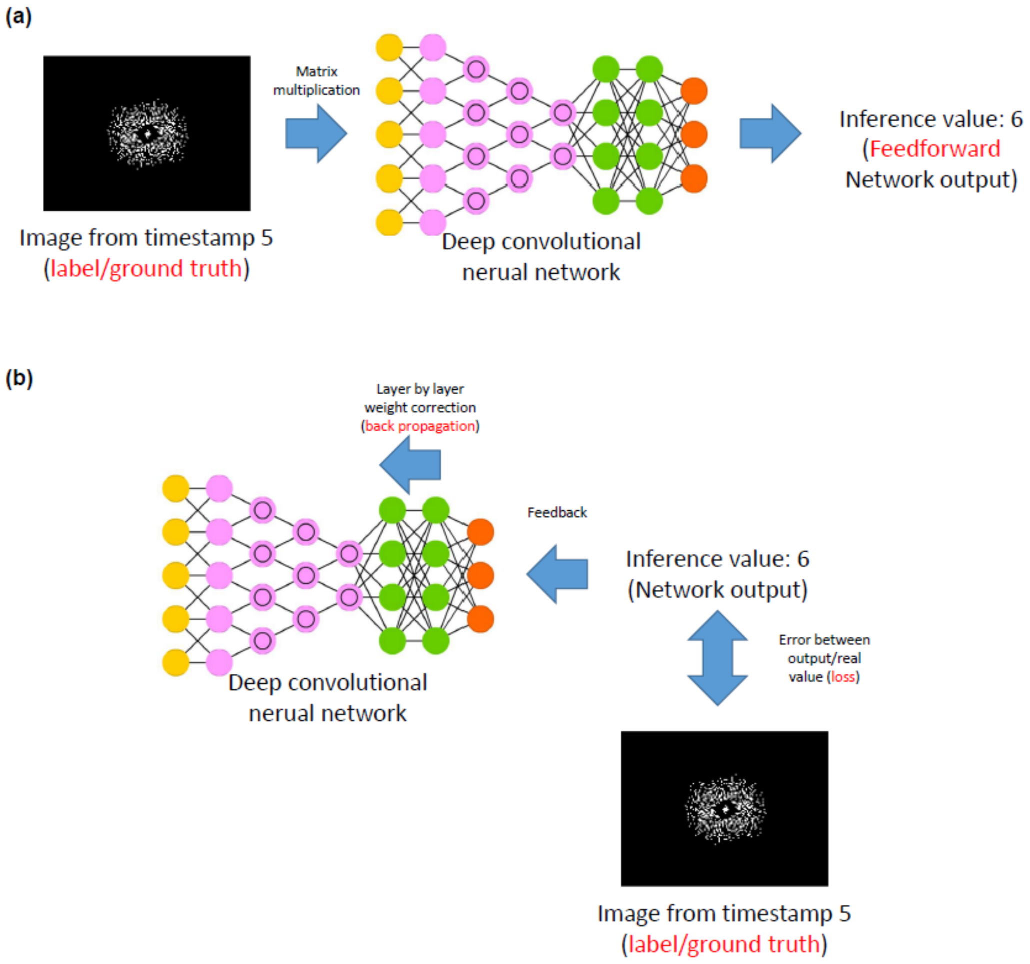

Supplementary Figure 4. (**a**) Explanary image showing feedforward inference of the Deep Convolutional Neural Network. (**b**) Explanary image showing the weight update of the neural network to decrease error between the real value and inferenced output. Important terms used in the main article were marked with red color.

References

1. Kim, K.; Choi, D.; Lim, H.; Kim, H.; Jeon, J. Vision Marker-Based In Situ Examination of Bacterial Growth in Liquid Culture Media. *Sensors* **2016**, *16*, 2179.
